# SILAC–based quantitative MS approach for real-time recording protein-mediated cell-cell interactions

**DOI:** 10.1038/s41598-018-26262-2

**Published:** 2018-05-31

**Authors:** Xixi Wang, Yu He, Yang Ye, Xinyu Zhao, Shi Deng, Gu He, Hongxia Zhu, Ningzhi Xu, Shufang Liang

**Affiliations:** 1 State Key Laboratory of Biotherapy and Cancer Center, West China Hospital, Sichuan University, and National Collaborative Innovation Center for Biotherapy, Chengdu, 610041 P. R. China; 2 Chengdu Center for Disease Control and Prevention, Chengdu, 610041 P. R. China; 3 Department of Urinary Surgery, West China Hospital, West China Medical School, Sichuan University, Chengdu, 610041 P. R. China; 4 Laboratory of Cell and Molecular Biology & State Key Laboratory of Molecular Oncology, Cancer Institute & Cancer Hospital, Chinese Academy of Medical Sciences, Beijing, 100021 P. R. China

## Abstract

In tumor microenvironment, interactions among multiple cell types are critical for cancer progression. To understand the molecular mechanisms of these complex interplays, the secreted protein analysis between malignant cancer cells and the surrounding nonmalignant stroma is a good viewpoint to investigate cell-cell interactions. Here, we developed two stable isotope labeling of amino acids in cell culture (SILAC)-based mass spectrometry (MS)/MS approaches termed spike-in SILAC and triple-SILAC to quantify changes of protein secretion level in a cell co-cultured system. Within the co-culture system of CT26 and Ana-1 cells, the spike-in SILAC and triple-SILAC MS approaches are sensitive to quantitatively measure protein secretion changes. Three representative quantified proteins (Galectin-1, Cathepsin L1 and Thrombospondin-1) by two SILAC-based MS methods were further validated by Western blotting, and the coming result matched well with SILACs’. We further applied these two SILACs to human cell lines, NCM460 and HT29 co-culture system, for evaluating the feasibility, which confirmed the spike-in and triple SILAC were capable of monitoring the changed secreted proteins of human cell lines. Considering these two strategies in time consuming, sample complexity and proteome coverage, the triple-SILAC way shows more efficiency and economy for real-time recording secreted protein levels in tumor microenvironment.

## Introduction

Tumor microenvironment, a complex system of many cell types including tumor, stroma, endothelial and immune system cells^1,2^, was recognized as the product of a developing crosstalk with secreted proteins elements. The secreted proteins in extracellular matrix (ECM), which produced by various cells in cell microenvironment, play very important roles in their growth and progression^2,3^. Proteins that are secreted into ECM, taken as a promising source of biomarkers^3,4^, also are responsible for signaling and communication in tumor microenvironment^4,5^. It provides new sights in cancer biology to pursue the dynamic changes of low-abundance secreted proteins in cancer microenvironment gives^6^.

The secretory pattern of tumor cells and their neighboring stromal cells is dynamically changed in tumor microenvironment. The secreted proteins are often present with high numbers and low abundance, and interfered by serum proteins in culture medium and contaminations of intracellular proteins released by cell lysis during sample preparation^7–9^. In order to sensitively detect active cell-cell interactions mediated by secreted proteins in tumor microenvironment, the SILAC (stable isotope labeling of amino acids in cell culture) based quantitative proteomic strategy showed us the good confidence due to its sensitivity and accuracy^6,10^. The SILAC-combined mass spectrometry (MS) has been widely used in varied studies, including screening disease biomarkers^9–13^, drug targets^14–16^, monitoring changes in post-translational modifications^17–19^ and finding key factors in the complex signal pathway^10,20,21^.

In classical SILAC-MS, two cell populations are respectively labeling with light, heavy isotope amino acids^10,13^, then cell lysates were combined to analyze together by LC-MS/MS. In the MS spectra, each isotope labeling peptide appears as a doublet with distinct mass differences. The differential protein abundances between two samples are calculated directly by comparing the intensity differences of the pair of isotope labeling peaks in MS^12,15^. So far, the typical SILAC-MS is only applicable for cell protein isotope labeling and quantification.

Lately, multiple SILAC-derived technical modifications have been developed to enlarge its practicability in protein quantification. For instance, the spike-in SILAC^22^, is developed in which the labeling is separated or ‘decoupled’ from the biological experiment. The non-labeled samples are combined with the SILAC standard, and each of these combined samples is analyzed separately by LC-MS/MS. The difference between the experimental samples is calculated as the ‘ratio of ratios’, where the ratio of one sample relative to the standard is divided by the ratio of the other relative to the standard. Actually, we previously have extended the SILAC-MS approach to tissue proteome comparisons, in which the heavy isotope labeling cells serve as the spike-in standards to compare the proteome changes of two states of tissues^20^.

Another similar strategy named super-SILAC method has been broadened five SILAC-labeling cell lines to serve as the internal standards for tissue proteome quantification^23^. Later, the triple-SILAC, namely SILAC with three isotope labeling states^24^, has been employed to characterize dynamic interaction partners in signal pathway. Generally, the multiple SILAC-based MS technique progresses greatly widen classic SILAC application in the study of clinical samples, secretome, post-translational modifications and organelle proteomes^25^.

Due to the complexity of multiple cell-involved tumor microenvironment, we aim to develop the spike-in SILAC or triple-SILAC approaches to real-time record protein-mediated cell-cell interactions. Based on our established SILAC methods^6,26^, we applied two cell lines in a co-culture system to simulate the tumor microenvironment, and adopted both the spike-in SILAC and triple-SILAC strategies to identify and quantify secreted protein changes. By comparing the feasibilities of these two methods, the triple-SILAC way shows more efficiency, economy for real-time recording secreted proteins in tumor microenvironment.

## Results

### Quantitative MS strategy for triple-SILAC and spike-in SILAC

**Figure 1:**
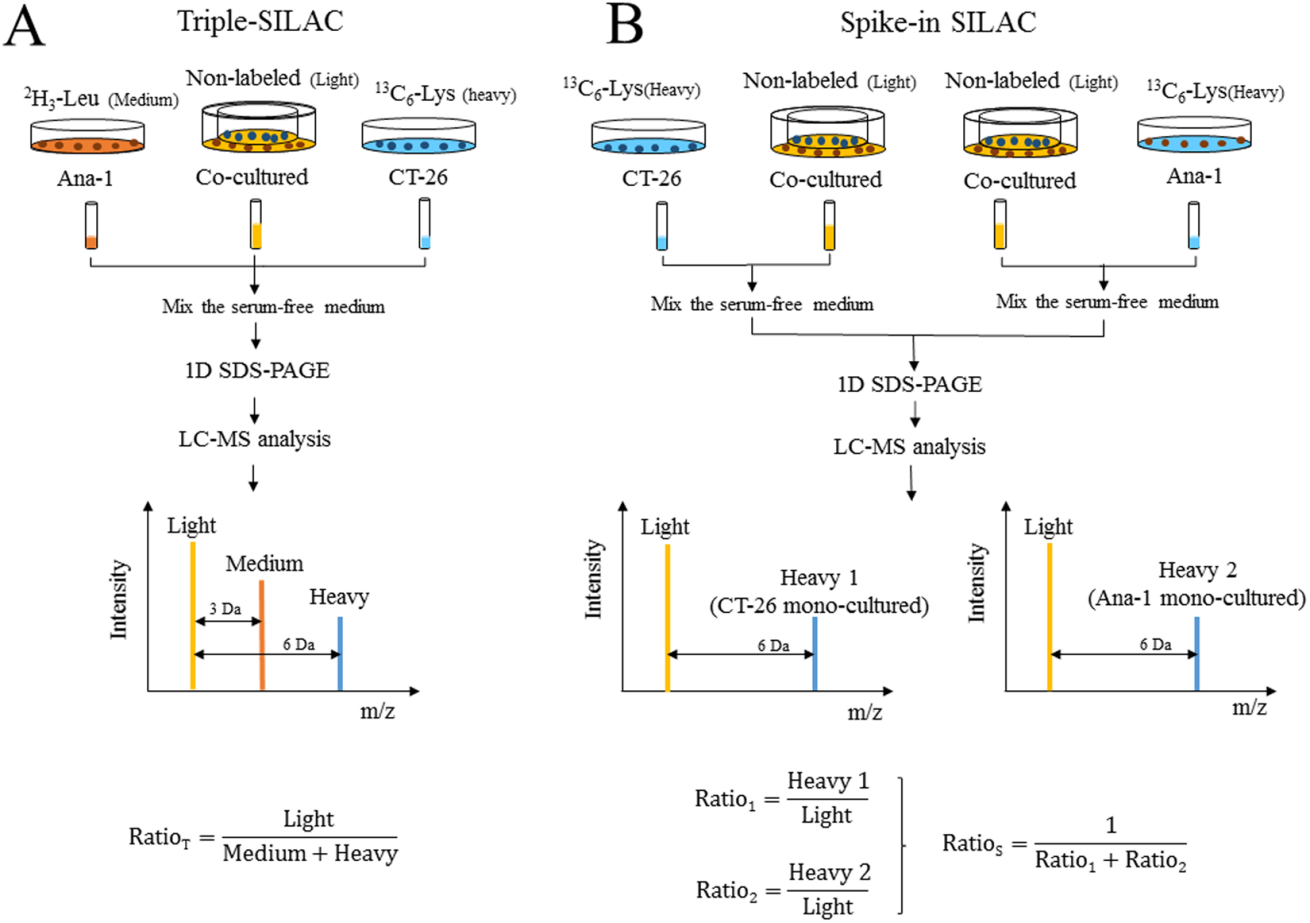
The strategies of ‘triple-SILAC’ (**A**) and ‘spike-in SILAC’ (**B**) approaches. In triple-SILAC, we combined the CMs from none labeled (L) co-cultured system, ^13^C_6_-Lys (H) labeled mono-cultured CT26 and ^2^H_3_-Leu (M) labeled Ana-1, then simultaneously analyzed by LC-MS/MS. The final ratio (Ratio _T_) of co-cultured system versus mono-cultured ones can be calculated directly by comparing the intensities of peaks with the certain molecular weight shifts in mass spectrum. In spike-in SILAC, CMs from ^13^C_6_-Lys (H) labeled Ana-1 and CT26 were combined separately with co-cultured one (L). To compare the peaks intensity with heavy (H) and light (L) labeled, Ratio1 and 2 were identified. The Ratio _S_ is the inverse of the sum of. Ratio1 and Ratio2. Light: non-labeled. Medium: ^2^H_3_-Leu labeled. Heavy: ^13^C_6_-lysine labeled. Ratio _T_ and Ratio _S_: Final ratio of a protein for co-cultured system versus mono-cultured in triple and spike-in SILAC. Ratio 1: the protein ratio of ^13^C_6_-Lys-labeling mono-cultured CT26 versus co-cultured cells. Ratio 2: the protein ratio of ^13^C_6_-Lys-labeling mono-cultured Ana-1versus co-cultured cells.

For triple-SILAC, cell culture supernatant collected from each state, including unlabeling (Light, L), labeling with ^13^C_6_-Lys (Heavy, H) or ^2^H_3_-Leu (Medium, M) was combined for a simultaneous comparison of protein abundances. The relative amount of proteins from different culture media (CM) can be calculated directly by comparing the intensities of the certain labeling peptide(s) peaks in MS. The SILAC ratio (Ratio _T_) was defined as the ratio of co-cultured system versus mono-cultured one. A certain secreted protein’s Ratio _T_ equaled to the unlabeling peptide(s) intensity divided by the sum of ^13^C_6_-Lys and ^2^H_3_-Leu labeling one(s) (Fig. 1A).

For spike-in SILAC, each ^13^C_6_-Lys labeling mono-cultured sample is separately combined with the co-cultured SILAC standard (none-label), which are then processed and analyzed together. Each of these two combined samples is analyzed separately by LC-MS/MS. The difference (Ratio _S_) between the experimental samples is calculated as the ‘ratio of ratios’, where the ratio of one sample relative to the standard is divided by the ratio of the other relative to the standard (Fig. 1B). The SILAC ratio1 was shown as the relative concentration of a secreted protein of ^13^C_6_-Lys-labeling mono-cultured cells versus co-cultured cells. Similarly, SILAC ratio2 was represented the relative protein amount of ^13^C_6_-Lys-labeling mono-cultured Ana-1 versus co-cultured cells.

In both SILAC-based strategies, it should be averaged when several peptides were used to quantify a protein. Due to uncertainty of the exact cell number we add in, the final ratios of both SILAC approaches should be normalized by total protein concentration in mono-and co-cultured CM.

### MS quantification analysis for secreted proteins

**Figure 2:**
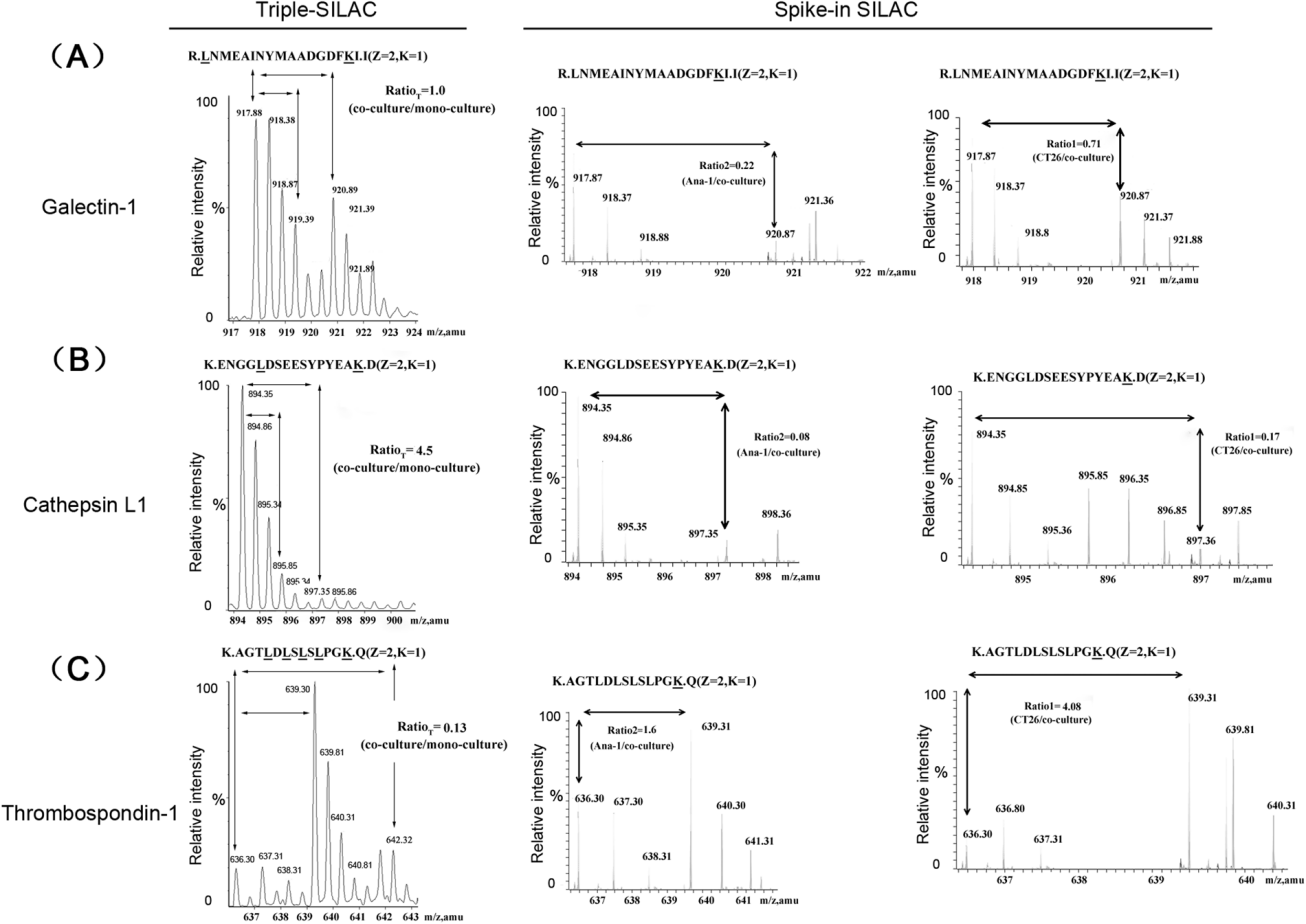
Quantitative MS peaks of three proteins identified by spike-in SILAC and Triple-SILAC in mouse Ana-1 and CT26 co-culture system. In triple-SILAC way, the MS spectra showed the peak intensity of the quantitative peptides with none (L), ^2^H_3_-Leu (M) and ^13^C_6_-Lys (H) labels. In spike-in SILAC, the quantitative peptide containing ^2^H_3_-Leu (M) and ^13^C_6_-Lys (H) labels were compared to none labeling (L) separately. In both ways, the monoisotopic peaks with highest intensity were taken to be compared. (**A**) The MS spectra of the quantitative peptide“R.LNMEAINYMAADGDFK.I” for Galectin-1. (**B**)The quantitative peptide “K.ENGGLDSEESYPYEAK.D” for Cathepsin L1. (**C**) The quantitative peptide “K.AGTLDLSLSLPGK.Q” for Thrombospondin-1.

CT26 and Ana-1 cells were co-cultured in a transwell system with 1:1 cell number ratio. To compare efficiency of triple-SILAC method with spike-in SILAC way, three identified proteins were representatively shown the quantitative results. For triple-SILAC, Cathepsin L1 was upregulated by 4.5 fold, and Thrombospondin-1 was down regulated by 0.13 fold in co-cultured system. The abundance was no change for Galectin-1. For spike-in SILAC, Cathepsin L1 increased by 2.86 fold (**Ratio**_**S**_), which was calculated by inversing the sum of Ratio1 and 2 (1/(0.17 + 0.18)) (Fig. 2B). Thrombospondin-1 was decreased by 0.18 fold (Ratio _S_ = 1/ (Ratio1 + Ratio2) = 1/ (1.60 + 4.08)) (Fig. 2C). The unchanged protein, Galectin-1, had a ratio at 1.07 (1/ (0.22 + 0.71) (Fig. 2A). In both strategies, these three proteins showed the same change trend (Table 1).

**Table 1:** Quantitative MS data of Galectin-1, Cathepsin L1 and Thrombospondin-1.

Protein	Triple-SILAC		Spike-in SILAC		
	Ratio _T_^a^ (n = 3)		Ratio1^b^ (n = 3)	Ratio2^c^ (n = 3)	Ratio _S_^d^
Galectin-1	1.00 ± 0.06		0.71 ± 0.13	0.22 ± 0.05	1.07
Cathepsin L	4.50 ± 1.37		0.17 ± 0.03	0.08 ± 0.02	2.86
Thrombospondin-1	0.13 ± 0.06		4.08 ± 0.31	1.60 ± 0.08	0.18

Notes: All quantitative data were recorded as mean ± SD.

^a^The mean SILAC Ratio _T_ equaled to the unlabeling peptide(s) intensity of co-culture divided by the sum of ^13^C_6_-Lys (the mono-cultured CT26) and ^2^H_3_-Leu (the mono-cultured Ana-1) labeling one(s).

^b^The mean SILAC ratio 1 of protein in the conditioned medium of mono-cultured CT26 versus the co-culture cells.

^c^The mean SILAC ratio 2 of protein in the conditioned medium of mono-cultured Ana-1 versus the co-culture cells.

^d^Ratio _S_ equaled to the reciprocal of the sum of ratio 1and ratio 2.

### Western blotting validation for SILAC-MS approaches

**Figure 3:**
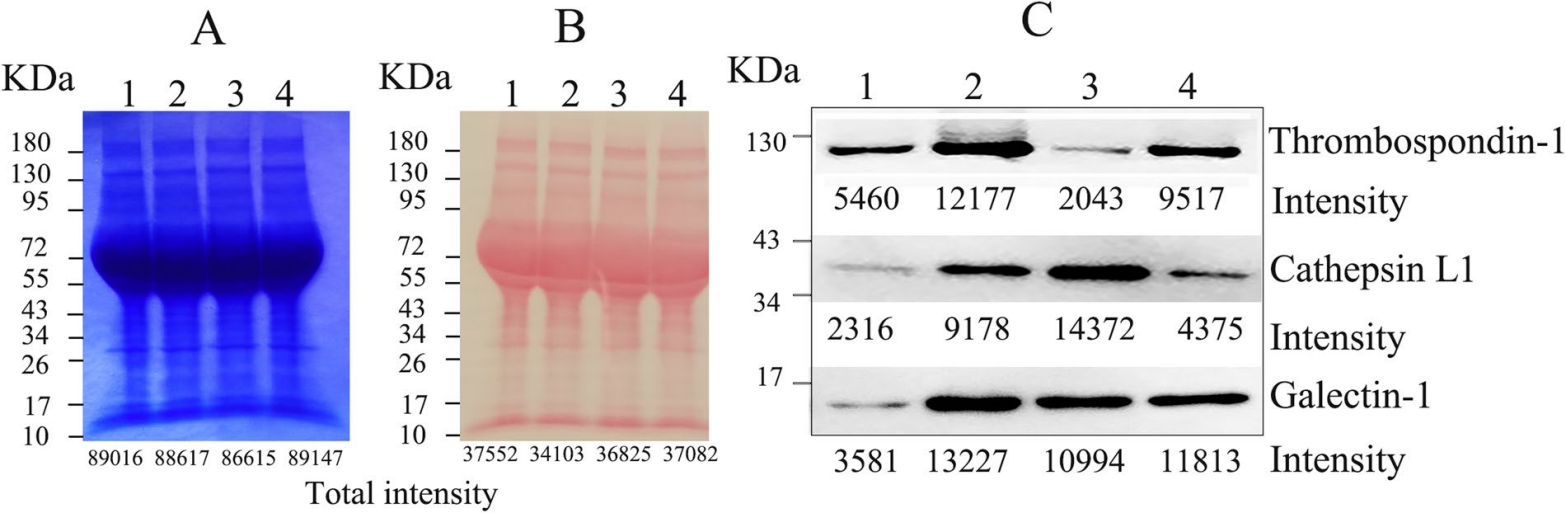
In-gel validation for three representative proteins. (**A**)The total proteins of loaded samples. (**B**) The total proteins after transferring to PVDF membrane. (**C**) The three MS- quantified proteins were verified by Western blot. Line 1–3: Conditional media (CM) collected from mono-cultured Ana-1 (1), CT26 (2) and co-cultured system (3). Line 4: the mixture CMs from mono-cultured Ana-1 and CT26 that have been incubated for 48 h respectively (4). Data showed the band intensity. The unprocessed original scans of blots (Fig. 3) are shown in Supplementary Fig. 1.

To validate the SILAC-MS quantification results, the three identified proteins were further detected by Western blotting in Ana-1/CT26 system (Fig. 3). The protein abundances were compared the band density using the Imagine J software (version 1.8.0)^27^. Firstly, to confirm the same loading amount of samples, we plotted the total proteins of 3 samples by Coomassie blue staining (Fig. 3A). Before immunoblotting, we also provided Ponceau S staining of the PVDF membrane to evaluate the efficiency of protein transfer from the SDS gel to membrane (Fig. 3B). The Gel analysis results showed Cathepsin L1 was upregulated to 3.29 fold (14372/4375). Thrombospondin-1 had lower intensity in co-cultured system versus mono-culture with a ratio of 0.21 (2043/9517). And Galectin-1 showed no obvious change with a ratio of 0.93 (10994/11813) in Western blotting analysis (Fig. 3C). The Western blotting results were highly consistent with the SILAC-MS quantification.

### The extensive application of SILACs in human cell line co-culture system

After successfully applying the two (SILAC)-based MS/MS approaches in CT26 and Ana-1 co-culture system, two human cell lines, NCM460 and HT29, were further chosen to validate the feasibility of analyzing secreted proteins in human colorectal carcinoma *in vitro*.

**Figure 4:**
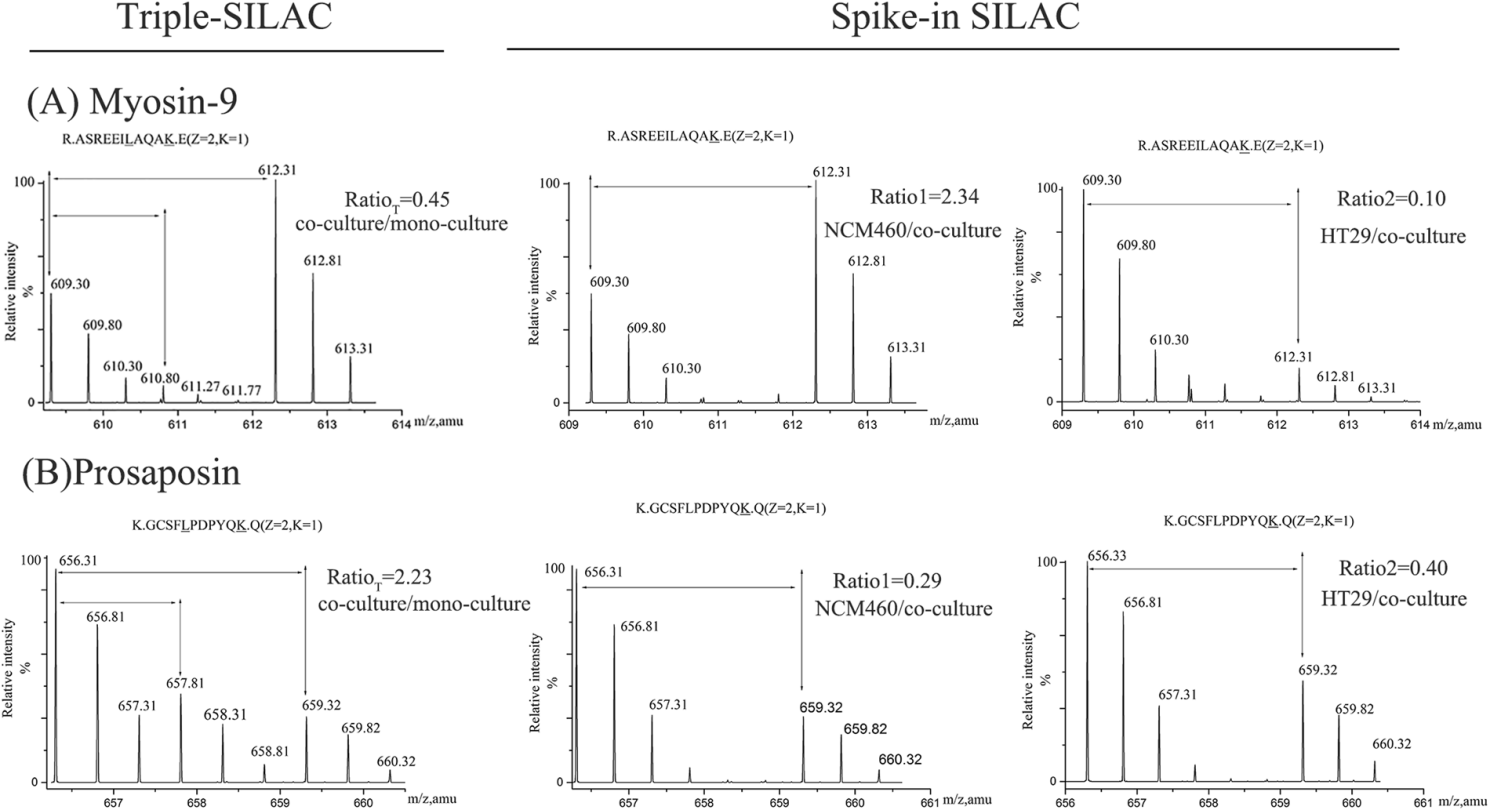
Quantitative MS peaks of two proteins identified by spike-in SILAC and Triple-SILAC in human NCM460 and HT29 co-culture system. (**A**) The MS spectra of the quantitative peptide “R.ASREEILAQAK.E” for Myosin-9. (**B**) The quantitative peptide “K.GCSFLPDPYQK.Q” for Prosaposin.

Similarly, we quantified two changed proteins in NCM460 and HT29 co-culture system using the above two (SILAC)-based MS/MS approaches. Two representative proteins, Myosin-9 and Prosaposin, were shown the quantification data (Fig. 4). Myosin-9 was respectively decreased by 0.45, 0.41 fold in triple and spike-in SILAC MS analysis for the co-culture system (Fig. 4A). Prosaposin (Psap), was identified to increase by the ratio of 2.23(Ratio _T_) in triple SILAC and 1.45 (Ratio _S_ = 1/ (Ratio1 + Ratio2) = 1/ (0.29 + 0.4)) in spike-in SILAC MS analysis (Fig. 4B). In both strategies, these two proteins showed the same trend (Table 2).

**Table 2:** Quantitative MS data of Prosaposin and Myosin-9.

Protein	Triple-SILAC	Spike-in SILAC		
	Ratio _T_^a^ (n = 3)	Ratio1^b^ (n = 3)	Ratio2^c^ (n = 3)	Ratio _S_^d^
Prosaposin	2.27 ± 0.16	0.33 ± 0.07	0.34 ± 0.11	1.53
Myosin-9	0.51 ± 0.17	2.74 ± 0.77	0.18 ± 0.06	0.38

Notes: All quantitative data were recorded as mean ± SD.

^a^The mean SILAC Ratio _T_ equaled to the unlabeling peptide(s) intensity of co-culture divided by the sum of ^13^C_6_-Lys (the mono-cultured NCM460) and ^2^H_3_-Leu (the mono-cultured HT29) labeling one(s).

^b^The mean SILAC ratio 1 of protein in the conditioned medium of mono-cultured NCM460 versus the co-culture cells.

^c^The mean SILAC ratio 2 of protein in the conditioned medium of mono-cultured HT29 versus the co-culture cells.

^d^Ratio _S_ equaled to the reciprocal of the sum of ratio 1 and ratio 2.

## Discussion

Each SILAC-MS method has individual strength and shortcoming (Table 3). For the triple-SILAC way, three samples are differentially labeled and combined before MS identification. The three-in-one sample just needs to process once, so the quantification deviation introduced between multiple unparalleled sample preparations can be eliminated. This also means we can apply as much steps to enrich proteins or peptides as we want to meet experiment demands. With a spike-in standard, each sample is measured separately, and therefore the measuring time is two to threefold longer than that for triple-SILAC experiments. In spike-in SILAC, the samples are processed and analyzed separately, the results are based on the ratios between the samples, in which it is dependent on the ratio of ratios, leading to an increase in the quantification variation. This effect will also take place when two triple-SILAC experiments are combined. The relative statistical error of a ratio of ratios is the square root of the sum of the squares of individual relative errors. Secondly, the biological issue and the system studied would be considered for Spike-in SILAC analysis. The makeup of the SILAC standard will vary depending on the complexity of the system of interest, ranging from single proteins to a complex mixture of thousands of proteins, and the approach is dependent on the variability of the samples. When there are more than three samples to be compared, triple-SILAC is out of our options.

**Table 3:** Advantages and disadvantages of two SILAC–based MS approaches.

Name	Description	Advantages	Disadvantages
spike-in SILAC	Decoupled mixtures of two kinds of labeling cells with a heavy/light spike-in standard	1. Tissues applicable 2. High coverage 3. Multiple samples comparing(n ≥ 3)	1. High quantification variation 2. Time consuming in sample processing & measuring 3. Limited sample multiplexing 4. Identify a proper spike-in standard based on target proteins and certain sample types.
Triple-SILAC	Mixture of three different labeled cell lines	1. Time saving 2. Low quantification variation 3.No need for SILAC standard	1. Not applicable to tissues 2. More complexity of sample and MS spectra 3. Comparisons for only three samples

Owing to the partial stochastic nature of shotgun proteomics, when the proteome coverage is low, the overlap between runs is also low, and it is difficult to compare samples. Naturally, the aim should always be to achieve high proteome coverage. This problem is irrelevant in triple-SILAC experiments, as all the peptides are directly compared in a single analysis. The complexity of the MS spectra is 1.5-fold higher in triple-labeled samples than that in spike-in standard experiments. High complexity may lead to lower numbers of identified proteins due to re-sequencing of peptides in the different SILAC states. But it is not observed in our co-cultured system which includes two cell lines.

The CT26 and Ana-1 both are mouse cell lines. The CT26 is a mouse colon fibroblast carcinoma cell line, and Ana-1 is a normal murine macrophage cell line. We chose these two cell lines for recording protein-mediated cell-cell interactions due to the following reasons. 1) Mouse cell lines are the mostly used in biological and medical research due to similarity with human cells in gene expression, ageing, and immune responses to infection and disease. 2) The secreted proteins in ECM are produced by various cells in cell microenvironment, which is dynamic to regulate cell growth and progression^2^. Tumor-associated macrophages are present in the stroma of many tumors, which are frequently associated with the progression of several types of cancer including colon cancer^6^. So CT26 and Ana-1 cell lines were selected to simulate colorectal carcinoma tumor microenvironment for developing and evaluating the power of SILAC-based MS approaches we applied in this paper.

In Ana-1 and CT26 system, totally 221 secreted proteins were quantified through spike-in SILAC-MS analysis, and 203 proteins were identified by triple-SILAC MS. The upregulated protein Cathepsin L1 in our co-cultured cells, is a lysosomal cysteine protease, which is overexpressed to secret into serum in several cancers, including ovarian cancer, pancreatic neuroendocrine cancer, glioma and others^28–30^. The overexpression of Cathepsin L1 is involved in tumor invasion, metastasis and chemotherapy resistance^31,32^. Thrombospondin-1 is a large glycoprotein secreted by platelets and synthesized by many cell types, including endothelial and tumor cells. Thrombospondin-1 may potentiate tumor progression, promotes tumor cell adhesion, migration and invasion^33^. The two changed secreted proteins are further explored their molecular functions between tumor cells and macrophages.

We further applied two (SILAC)-based MS/MS approaches to validate the feasibility of analyzing secreted proteins in human NCM460 and HT29 system *in vitro*, which confirmed the spike-in and triple SILAC were capable of monitoring the changed secreted proteins of human cell lines (Fig. 4).

The original SILAC labeling is limited to cell culture or microorganisms. To extend this powerful technique to higher organisms, Hayter and colleagues demonstrated chicken can be partially labeled at the amino acid level by feeding them with a diet containing stable isotope-labeled valine^34^. Drosophila can be labeled by feeding the flies with ^13^C_6_-Lys labeled yeast. Krueger et.al achieved the ^13^C_6_-Lys labeling laboratory mouse^35^. Until now, this so-called ‘SILAC mouse’ is the only multicellular organism that has been completely labeled with the SILAC approach and partial labeling was recently achieved in newts^34,36^.

SILAC-labeled mice are generated by feeding them with ^13^C_6_-Lys containing diet. Although labeling rates reach 95% which is required to perform comparative and quantitative proteomics by MS after two generations. This is both costly and time-consuming and as a result it hampers repeated generation of labeled organisms for each experimental condition, each strain or transgenic organism. Different cell types and tissues in the same organism have different SILAC label incorporation, and extending the labeling time cannot perform complete labeling due to the recycling of internal amino acid sources^35^. This may lead to the quantification uncertainty.

It is recommended using the SILAC model organisms just as standards rather than as the experimental system themselves, since the SILAC food might have metabolic effects^35^. Moreover, with the spike-in standard approach the same SILAC organism can be used for experiments with strains of various genetic backgrounds. In our co-culture system, we use the unlabeled co-culture cells as the spike-in standard.

For triple SILAC MS application on animal models, we need to build two different labeled model animals, which is time consuming and much more cost-expensive. Besides lysine, there are limited peptides are suited for the whole organism labeling now. Arginine is also problematic since it is converted into proline in some cell lines^37^. Leucine is one of the most used amino acid for SILAC labeling, but it is not the tryptic-digested peptide end, which is not optimal for global or large-scale SILAC quantitation^12^.

^15^N is also used to label entire rats, particularly for quantitative brain proteomics^38^. Despite its usefulness, ^15^N labeling has also several disadvantages. Since most peptides contain dozens of nitrogen atoms, labeling with highly enriched ^15^N still results in only partial peptide labeling and therefore complex isotope clusters. In addition, the mass shift between the labeled (i.e. heavy) and unlabeled (i.e. light) form of a peptide depends on the number of nitrogen atoms and therefore varies depending on the peptide sequence. This leads to an increase in the number of candidate masses that need to be considered and therefore complicates peptide identification by search algorithms. Both problems result in smaller identification rates and less accurate quantification that can partially overcome by computational correction^39^.

## Conclusion

The SILAC-based quantitative proteomic techniques, including the spike-in SILAC and triple-SILAC, are successfully applied to identify secreted protein changes in mimic tumor microenvironment *in vitro*. Considering the availability of these two methods, the triple-SILAC MS shows more efficiency, economy for real-time recording secreted protein levels in tumor microenvironment.

## Methods

### Chemicals and reagents

Fetal bovine serum (FBS) (AUS-01N, CELL-BOX), RPMI 1640 media (Gibco Gaithersburg), Dulbecco’s Modified Eagle’s Medium (DMEM) (Gibco, Gaithersburg) ^13^C_6_-lysine (98%; 89988, Thermos), and ^2^H_3_-leucine (DLM-1259-1, 99%; Cambridge Isotope Laboratories). Coomassie blue and Ponceau S (GOLDBIO). The other reagents for MS analysis were purchased from Sigma-Aldrich (St. Louis, MO) unless otherwise stated.

### Cell culture

CT-26, a mouse colon fibroblast carcinoma cell line, and a normal murine macrophage cell line Ana-1 were both ordered from American Type Culture Collection (ATCC). Cells were cultured in the RPMI-1640 Medium, supplemented with 10% FBS at 37 °C in humidified atmosphere with 5% (v/v) CO_2_.

The NCM460, a normal human colon mucosal epithelial cell line, was ordered from Incell Corporation, LLC (www.incell.com). HT29, a colon adenocarcinoma cell line, was ordered from ATCC. These cells were stored in our laboratory^40^. Cells were routinely maintained in Dulbecco’s Modified Eagle’s Medium (DMEM) (Gibco, Gaithersburg, MD) supplemented with 10% FBS at 37 °C in humidified atmosphere with 5% (v/v) CO_2_.

### Stable isotope labeling

CT-26 cells were cultured in RPMI 1640 media with 0.1 mg/ml ^13^C_6_- Lys replacing normal Lys. Meanwhile Ana-1 cells were cultured in RPMI 1640 media with 0.1 mg/ml ^13^C_6_- Lys or ^2^H_3_-Leu to respectively replace normal Lys or Leu.

NCM460 cells were cultured in DMEM with 0.1 mg/ml ^13^C_6_- Lys replacing normal Lys. Meanwhile HT29 cells were cultured in DMEM media with 0.1 mg/ml ^13^C_6_- Lys or ^2^H_3_-Leu to respectively replace normal Lys or Leu.

All Cell lines were cultured and routinely maintained at 37 °C in humidified air containing 5% CO_2_. The culture media were changed every 2–3 days. Cells were kept in ‘labeled’ medium for at least 5 cell passages to achieve >95% labeling.

### Co-culture conditions

To modulate the interactions between the two kinds of cell lines, Ana-1 and CT26 cells were co-cultured on a transwell system (3419, Corning Inc., Corning, NY) with 1:1 cell number ratio. In co-culture system, 5 × 10^6^ unlabeling Ana-1 cells were seeded into the bottom chamber of a 75mm-transwell plate and incubated in 8 ml serum-free RPMI 1640, meanwhile the same amounts of unlabeling CT26 cells were also cultured in 8 ml serum-free RPMI 1640 onto the top chamber of the transwell plate. Cells were co-cultured for 48 h.

For NCM460 and HT29 cells, the co-cultured system is just as same as Ana-1 and CT26 in 8 ml serum-free DMEM, with NCM460 cells in the bottom chamber and HT29 in the top one.

In triple-SILAC way, the same amount of ^2^H_3_-Leu labeled cells (Ana-1 or HT29) and ^13^C_6_-Lys cells (CT26 or NCM460), as the mono-culture system, were cultured in 8 ml serum-free ^2^H_3_-Leu and ^13^C_6_-Lys added-in RPMI 1640 or DMEM for 48 h respectively. For spike-in, ^13^C_6_-Lys labeled cells (Ana-1, CT26, NCM460 and HT29) were cultured in ^13^C_6_-Lys containing RPMI 1640 or DMEM.

### Secreted protein preparation

Secreted protein preparation was performed according to our previous method^6^. Briefly the conditioned medium (CM), which separately derived from co- and mono- culture system in triple-SILAC and spike-in SILAC strategies, was collected by centrifugation at 1500 × g for 3 min to remove cell debris following with a sterile filtration (0.22μm, Millipore). With an addition of proteinase inhibitor (cocktail,P-8340,sigma), the CM was concentrated by ultrafiltration using a centrifugal filter device “AmiconUltra-15” (UFC900324, Millipore) with centrifugation at 4000 × g for 2 h. Proteins were precipitated with cold acetone at −20 °C overnight and collected by centrifugation at 13,000 g for 20 min at 4 °C.The protein pellet was air-dried at room temperature, and resolved in 40 μL sample buffer (60 mM Tris-HCL pH 6.8, 2% SDS, 10% glycerol, 5% DTT, 0.01% bromophenol blue). The protein concentration was determined by Bradford protein assay (P0011, Beyotime).

### Sample combination

The CMs were mixed from the different cultured cell system. In triple-SILAC mode, we mix all three CMs into one part, which contains non-labeled (light), ^2^H_3_-Leu (medium) and ^13^C_6_-Lys (heavy) labeled proteins. For spike-in, the different labeled mono-cultured CM was combined with co-cultured one respectively.

### Proteins in-gel digestion

Proteins were separated by 12%SDS-PAGE and stained with coomassie to visualize gel bands. The gel lanes were excised to cut into 1 mm^3^ slices and destained twice with 100 mM NH_4_HCO_3_/50% acetonitrile (ACN) at 37 °C for 30 min. After being dehydrated and dried, gel slices were digested with 10–20 μl MS-grade trypsin (V5280, Trypsin Gold, Promega, USA) at 37 °C overnight. Peptides were extracted by ultrasonic with extraction solution (50%ACN/5% trifluoroacetic acid) for twice. The combined extraction was dried in a vacuum concentrator at room temperature. Samples were then subjected to LC-MS/MS analysis.

### LC-MS/MS analysis

Proteins were identified by nanoLC-MS/MS with LTQ-Orbitrap (Thermo Finnigan, San Jose, CA). Peptide samples were separated on a C_18_ column (150 μm, 150 mm length, Column Technology Inc.) after being desalted on a trap column (Zorbax 300 SB C_18_, Agilent Technologies, Palo Alto, CA). The mobile phase A was 0.1% formic acid in HPLC-grade water, and 0.1% formic acid in ACN for the mobile phase B. The peptide mixture was separated at 2 μl/min with a linear gradient of 4%–50% B for 110 min, following by 50%–100% B from 110 min to 115 min, then maintained at 100% B for 5 min. Data dependent MS/MS mode was acquired, in which each scan cycle consisted of one full MS scan in profile mode followed by seven MS/MS scans in centroid mode.

### Mass data search

The Mass data were processed by Maxquant software 1.6.0.1 plugging Andromeda 1.5.6.0 as the database search engine. The parameters for database searching were set as following: database, Swiss-Prot databases (16523 proteins (Mouse, Aug 30, 2017) and 43943 proteins (Human, Oct 13, 2017)); enzyme, trypsin; two missed cleavages are allowed. Carbamidomethyl (C) was selected as a fixed modification, and oxidation (M), acetyl (protein N-term), ^13^C_6_-Lys and ^2^H_3_-Leu labeling (only for triple-SILAC) were set as the variable modification. Initial peptide mass tolerance was set to 7 ppm and fragment mass tolerance was 0.5 Da. + 2 as default charge state of each peptide. The false discovery rates (FDRs) of peptide and protein were both set to 0.01, which ensure that protein identifications with FDRs ≤ 1% (95% confidence). At least one unique peptide of a protein successfully detected was considered to be acceptable.

### Coomassie blue and Ponceau S staining

The SDS-PAGE gel was fixed in solution (50% methanol and 10% glacial acetic acid) for 2 hours with gentle agitation. It was stained in staining solution (0.1% Coomassie Brilliant blue R-250, 50% methanol and 10% glacial acetic acid) for 2–3 hours with gentle agitation. After destaining in solution (40% methanol and 10% glacial acetic acid) for several times until no unspecific background, the gel was stored in 5% glacial acetic acid.

The blotted membrane was immersed in a sufficient amount of Ponceau S staining solution (0.1% (x/v) Ponceau S in 1% (v/v) acetic acid) to stain for 5 minutes. Then membrane was immersed in an aqueous solution containing 5% acetic acid (v/v) for 5 minutes to evaluate the efficiency of protein transfer from the SDS gel to PVDF membrane.

### Western blot

We choose three typical secreted proteins (Cathepsin L1, Galectin-1 and Thrombospondin-1) to validate by Western blot analysis. Besides three identified proteins in SILACs changed in different direction: no change, up-and down regulation.

It was totally same in the way of cell culturing and protein extraction just as described above with the only difference that all culture system was made of non-labeling serum-free RPMI1640 medium, no ^2^H_3_-Leu or ^13^C_6_-lys. The protein concentration was determined with the Bradford protein method. 40 μg protein was separated on a 12% SDS-PAGE gel and transferred onto a PVDF membrane at 100 V for 1 h. Subsequently the membrane was incubated in TBST buffer (20 mM Tris–HCl, pH 7.6, 150 mM NaCl, 0.1%Tween-20) with 5% non-fat milk at room temperature for 2 h. The specific primary antibodies were diluted in TBST (50 mM Tris–HCl, with 150 mM NaCl,0.1%Tween-20, pH7.4) buffer to incubate PVDF membrane at 4 °C overnight. Primary antibodies included mouse anti-Galectin-1 (1:200, sc373717, Santa-Cruz, CA), mouse anti-Thrombospondin-1 (1:200, sc59886, Santa-Cruz, CA), and mouse anti-Cathepsin L1 antibodies (1:200, sc13094, Santa-Cruz, CA). The corresponding mouse derived secondary antibody conjugated horseradish peroxidase was subsequently incubated with the PVDF membrane for 60 min at room temperature. Signal detection was performed with ECL reagent (Amersham Biosciences, Piscataway, New Jersey, USA).

### Data availability statement

The datasets generated during and/or analyzed during the current study are available from the corresponding author on reasonable request.

## Electronic supplementary material

Supplementary data file
