# Supplementary material for: SILAC–based quantitative MS approach for real-time recording protein-mediated cell-cell interactions
Source: Sci Rep. 2018 May 31;8:8441. doi: 10.1038/s41598-018-26262-2 (PMC5981645; doi:10.1038/s41598-018-26262-2)
Supplement: Supplementary file 1 — Supplementary data file [file 41598_2018_26262_MOESM1_ESM.doc]

**SILAC–based quantitative MS approach for real-time recording protein-mediated cell-cell interactions**

Xixi Wang^1,2^ , Yu He^1^, Yang Ye^1^, Xinyu Zhao^1^, Shi Deng^3^, Gu He^1^, Hongxia Zhu^4^, Ningzhi Xu^1,4^, Shufang Liang^1*^

^1^ State Key Laboratory of Biotherapy and Cancer Center, West China Hospital, Sichuan University, and National Collaborative Innovation Center for Biotherapy, Chengdu, 610041, P. R. China

^2^ Chengdu Center of Disease Control and Prevention, Chengdu, 610041, P. R. China

^3^ Department of Urinary Surgery, West China Hospital, West China Medical School, Sichuan University, Chengdu, 610041, P. R. China

^4^ Laboratory of Cell and Molecular Biology & State Key Laboratory of Molecular

Oncology, Cancer Institute & Cancer Hospital, Chinese Academy of Medical Sciences, Beijing, 100021, P. R. China

*Correspondence to Dr. Shufang Liang (E-mail: [zizi2006@scu.edu.cn](mailto:zizi2006@scu.edu.cn)).

**Supplementary data file**

**Contents:**

Legend of the supplementary figure 1

Supplementary figure 1

Supplementary Figure1. The original loading proteins were visible on SDS-PAGE gel by Coomassie blue staining (A), and proteins on SDS-PAGE gel were transferred to the PVDF membrane to stain by the Ponceau S dye (B). C was shown the original Western blot profiling of the three proteins on the PVDF membrane.

A B


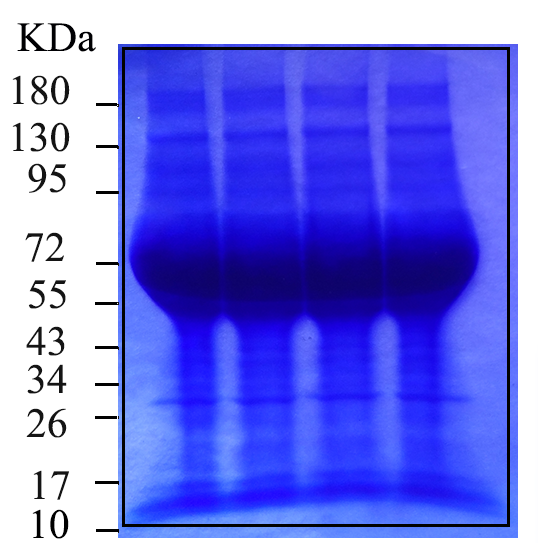

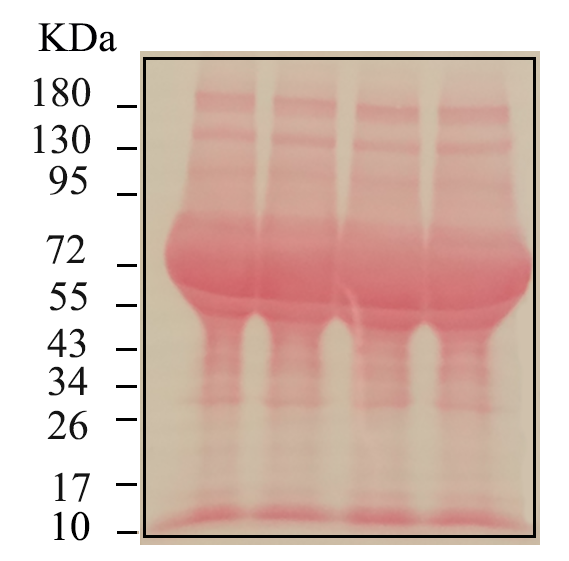


Total protein

C


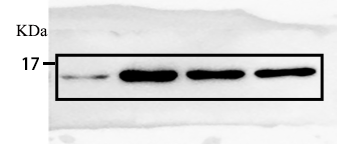

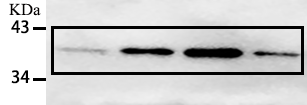


WB: Galectin-1 WB: Cathepsin L1


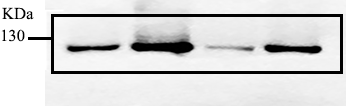


WB: Thrombospondin-1
